# Phased Return of Students to 77 Transitional Kindergarten-8th Grade Schools With Cohesive Mitigation Strategies Serving as Protective Factors Against the Increase of COVID-19 Cases in Marin County: September 2020-January 2021

**DOI:** 10.7759/cureus.19821

**Published:** 2021-11-22

**Authors:** Shayne Q Paff, Rochelle Ereman, Lisa Santora, Bethany Dominik, Alana McGrath, Jasmine Soriano, Karina Arambula, Charis Baz, Matthew Willis, Michaela F George

**Affiliations:** 1 Epidemiology and Public Health, Marin County Department of Health and Human Services, San Rafael, USA; 2 School of Public Health, Baylor University, Waco, USA; 3 Whole Person Care, Marin County Department of Health and Human Services, San Rafael, USA; 4 Global Public Health, Dominican University, San Rafael, USA

**Keywords:** pediatric quarantine/isolation, public health, school health, education equity, covid-19 school protocols, effective mask wearing, in-person learning, safe school reopening

## Abstract

Background and objective

Earlier uncertain implications of the coronavirus disease 2019 (COVID-19) pandemic on the pediatric population prompted the authorities to close schools worldwide under the premise that school settings would serve as drivers of an increase in the cases of COVID-19. Safe and equitable full-in-person school instruction is a critical factor in the continued educational gains of children and for their general well-being. The objective of this study was to report epidemiological trends related to the increasing percentage of students returning to in-person instruction, the suspected in-school transmission of severe acute respiratory syndrome coronavirus 2 (SARS‐CoV‐2), the virus that causes COVID-19, and countywide COVID-19 case rates during the first 21 weeks of school reopening in Marin County, CA, in the fall of 2020.

Materials and methods

The institutional review board (IRB) approval was waived for this study as it did not involve any identifiable human subjects data. Retrospective electronic reviews of countywide COVID-19 daily case count and COVID-19-related reports associated with in-person school participants from 77 schools in Marin County, CA, from September 8, 2020, to January 29, 2021, were conducted. The data were made available in collaboration with the Marin County Office of Education (MCOE) and Marin County Department of Health and Human Services (Marin HHS). Descriptive trends analyses were performed to determine whether the phased increase of students attending in-person learning was a significant contributor to countywide COVID-19 incidence rate, crude rate, and in-school COVID-19 viral transmission. This is the first long retrospective study of COVID-19 data among the reopened school population during the second half of the first pandemic year. It was conducted in a 21-week surveillance period involving an immense collaboration between Marin County’s public health officials and school administrators.

Results

Over the 21-week observational period involving 17,639 students, 4,938 school staff, and 899,175 student days, the countywide COVID-19 crude rate decreased (from 89.9 to 35.89 per 10,000) as more students returned to in-person learning. The schools’ strict adherence to public health guidance and site-specific safety plans against COVID-19 yielded a significantly reduced incidence rate of 0.84% among in-person learning participants; only nine cases were traced to suspected in-school SARS-CoV-2 transmission by way of rigorous contact tracing. The countywide COVID-19 incidence rate was 2.09%.

Conclusions

It is possible to minimize COVID-19 transmissions in in-person learning settings with cohesive mitigation strategies, specifically strict adherence to proper masking by students and staff while on school grounds. There is no clear correlation that the increasing phased return of students to in-person school drove an increase in countywide COVID-19 cases in Marin County, CA. Our findings revealed that schools were capable of safely resuming operations by following public health orders and recommendations. The increasing percentage of students returning to in-person school did not drive an increased COVID-19 case rate in the community. On the contrary, this analysis revealed that there was a drop in countywide COVID-19 cases as the phased student return percentage increased.

## Introduction

The novel severe acute respiratory syndrome coronavirus 2 (SARS-CoV-2) that caused the coronavirus 2019 (COVID-19) pandemic led to the interruption of education systems worldwide and removed an essential pillar of support for education and childhood well-being [1-3]. Rapidly evolving COVID-19 uncertainties prolonged the closure of in-person learning in the United States since March 2020, disproportionately affecting families of lower socioeconomic status [4], single parents, and those with special-needs children [5]; this further exacerbated disparities in education delivery, and has infused anxiety among all stakeholders involved, especially among educators [6], regarding the safety of school reopening.

Since the fall of 2020, several public schools in the United States [7-9] and across the world [9-11] have safely resumed in-person learning, while many private schools and select special education learning centers have continued to operate on approved in-person instruction waivers. The studies [7-11] pertaining to these schools have reported that with the right mitigation measures on school sites, in-person learning can be safely conducted, leading to only minimal in-school transmission of the disease at worst. They have concluded that in-person learning participants, with compliance to proper masking, physical distancing, and stable cohort strategies did not drive widespread SARS-CoV-2 transmission [7-11]. Additionally, a South Korean study [12] of hospitalized children (median age of seven years) diagnosed with COVID-19 and their caretakers reported no child-to-adult SARS-CoV-2 transmission with proper masking, further adding to the evidence that personal protective equipment (PPE), such as well-fitted two to three ply fabric or surgical masks, is effective at mitigating COVID-19 spread and that child-to-adult SAR-CoV-2 transmission is a rare occurrence [13]. Furthermore, previous studies [14] have reported that adult-to-child transmission occurred more prevalently in settings where there is household mixing of adults in the absence of proper use of face coverings, physical distancing, and adequate ventilation.

This research report describes the protective trends observed over a 21-week period among 77 reopened Transitional Kindergarten-8th Grade (TK-8) Schools and special education schools in Marin County, CA, that adhered to site-specific safety plans and involves 17,639 students [15] and 4,938 school staff [15] who participated in in-person learning for 899,175 student days [16]. We aim to highlight how the school reopening correlated with lower countywide COVID-19 case rates as more students returned to school sites for in-person learning over the 21-week surveillance period. We also emphasize the need for timely and continued dissemination of evidence-based information on safety feasibility [17] for in-person learning on school sites, with the purpose of alleviating the escalating anxiety among educators and families regarding the resumption of full in-person instruction at schools. We engage in a discussion on the SARS-CoV-2 Delta variant with reference to a published case report on an outbreak in a small school where an unvaccinated teacher’s noncompliance with consistent masking protocol resulted in multiple in-school transmissions of the COVID-19 virus.

This article was previously presented as a meeting abstract at the 2021 Meeting of the Society for Epidemiologic Research on June 23-25, 2021.

## Materials and methods

A week-to-week observational trends analysis was performed to investigate whether the phased return of TK-8 students correlated with an increase in countywide COVID-19 cases. Data used in the analysis was made available in collaboration with the Marin County Office of Education (MCOE) and Marin County Department of Health and Human Services Epidemiology COVID-19 Surveillance (Marin HHS COVID-19 Surveillance), Marin County Case Investigation/Contact Tracing (CI/CT), Marin County School Dashboard (MCSD), and Schools Data Team (SDT). The observational study period began on September 8, 2020, as schools in Marin County, CA, started a phased resumption of in-person learning, adopting county and state guidelines [17-18] for safe school reopening. The study period involved a 21-week observational period of countywide and school-related COVID-19 cases. It included a sample population of 17,639 students and 4,938 school staff who participated in in-person learning for a duration of 899,175 student days. The data collected only pertain to the surveillance period starting from September 8, 2020, through January 29, 2021. Data from the mentioned sources were extrapolated from the official Marin County Health and Human Services (Marin HHS) and MCOE websites. The access to view the Excel spreadsheet and Google Sheet for data collection was granted by the designated entities from each organization. Correspondence with MCOE and Marin HHS epidemiologists, public health officers, and public health nurses confirmed the credibility of the collected data.

COVID-19 case was defined based on the California Department of Public Health (CDPH) standards, as a laboratory test result confirmed through polymerase chain reaction (PCR) testing. A school-related case was defined as a COVID-19 case in a student or school staff involved in in-person learning. All school-related cases underwent thorough case investigation and contact tracing. A case investigator and contact tracer from Marin HHS gathered information about the school-related positive case, including close contacts, whereabouts and activities, and compliance with public health orders and recommendations. A suspected in-school transmission was defined as an event where SARS-CoV-2 was most likely transmitted between individuals while on school grounds. Countywide COVID-19 case counts were based on routine county-level reporting of daily laboratory-confirmed (by PCR testing) COVID-19 cases.

Marin County public health officials and school district administrators developed a 30-point plan [15] for safe school reopening, which was instituted as the guideline for school districts to reopen for in-person learning. Marin County public health officials also developed a team dedicated to keeping schools open throughout the first year of the pandemic. Each school was required to designate a public health liaison to be abreast of current and rapidly evolving public health guidance for safe school operations. Weekly webinars between Marin County public health officials and school administrators were instituted to provide support and guidance. Face covering was required for all in-person learning participants. Students in TK to 2nd grade were supported and taught how to properly wear a face mask. Certain outdoor activities on school grounds were scheduled for students to briefly remove face coverings. Meals were individually plated or bagged and served outside as deemed feasible or in classrooms by dividing students into cohorts. Physical distancing of six feet was observed in the classroom. All essential workers on school grounds were required to adhere to these guidelines. Non-essential school visits during operational hours were restricted.

## Results

School-related COVID-19 incidence rate was found to be lower (0.84%; 189 cases among 22,571 persons) than the countywide COVID-19 incidence rate (2.09%; 5,450 cases among 260,814 persons) [18]. Marin HHS Schools Tally data sheet [19] reported nine of the 189 school-related cases as suspected in-school transmissions: seven among students, two among school staff. No child-to-adult SARS-CoV-2 transmission was reported to have occurred on school sites per CI/CT, with the term adult referring to school staff. These results are descriptive accounts of all COVID-19 cases in Marin County from September 8, 2020, to January 29, 2021.

**Figure 1:**
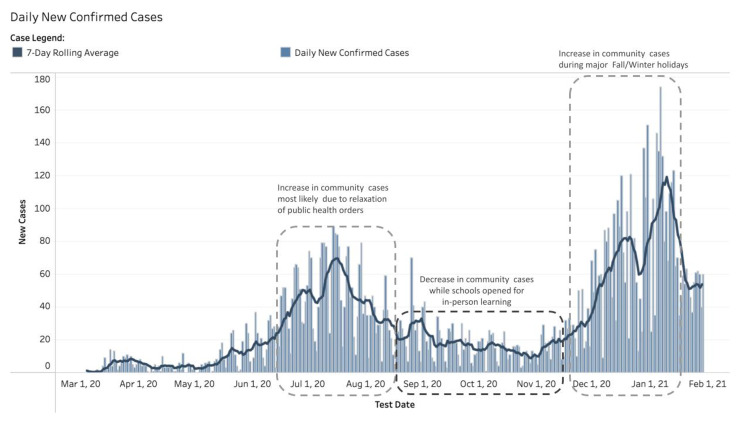
Countywide COVID-19 cases in Marin County, CA The chart shows a high incidence of COVID-19 community cases from July 2020 to August 2020. In the following 12 weeks after the first 73 schools reopened for in-person learning, COVID-19 community cases decreased. An exponential increase of COVID-19 cases occurred during the period from November 30, 2020, to January 8, 2021. A steep decline in COVID-19 cases occurred during the period from January 11, 2021, to January 29, 2021. Figure 1 acknowledgment: County of Marin HHS COVID-19 Surveillance website COVID-19: coronavirus disease 2019

A high incidence of COVID-19 community cases was observed between July 2020 and August 2020. In the following 12 weeks after the first 73 schools reopened for in-person learning, the countywide COVID-19 community cases decreased as well. An exponential increase of COVID-19 community cases occurred during the period from November 30, 2020, to January 08, 2021, followed by a steep decline of COVID-19 community cases during the period from January 11, 2021, to January 29, 2021 (Figure 1).

**Figure 2:**
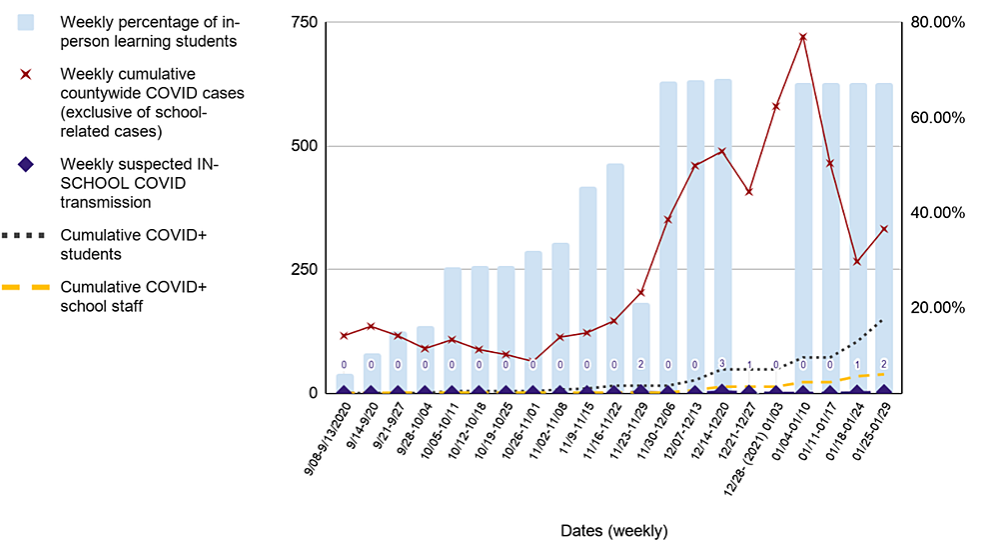
School data and countywide COVID-19 cases in Marin County COVID-19: coronavirus disease 2019

A week-to-week trends analysis shown in Figure 2 compares countywide COVID-19 cases, percentage of in-person learning students, weekly cumulative school-related COVID-19 cases, and suspected in-school transmissions. A steady decline in countywide COVID-19 cases began two weeks after the initial school reopening phase. From November 23, 2020, to November 29, 2020, the percentage of in-person learning students declined from 50.4% in the previous week to 21.14%. Countywide COVID-19 cases began to exponentially increase during that same week, which continued for three weeks despite the steady percentage of in-person learning students returning to schools. A momentary decline of countywide COVID-19 cases was observed from December 21, 2020, to December 27, 2020, the week of the winter holidays. An exponential increase for two weeks (December 28, 2020, through January 10, 2021) ensued soon after in the absence of in-person learning from December 21, 2020, to January 3, 2021. A steady decline of countywide COVID-19 cases was observed as in-person learning participants returned to school sites at a constant rate after the winter holidays.

**Figure 3:**
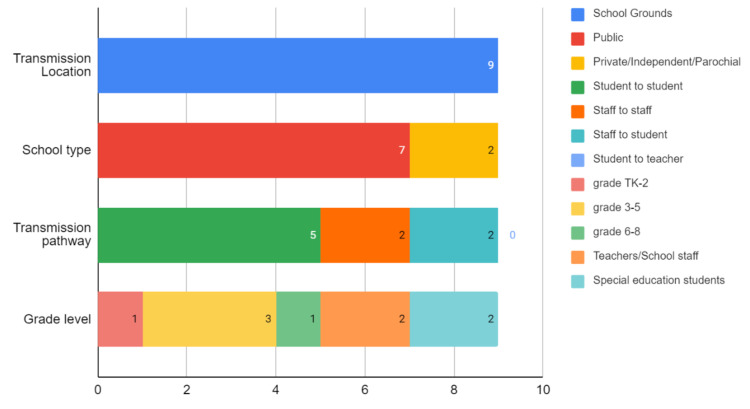
Descriptive statistics of nine suspected in-school transmissions

Figure 3 shows the breakdown of suspected in-school transmission cases. Of the nine suspected in-school transmission cases [19] to have occurred on school grounds, seven were from public schools, and two were from private/independent or parochial schools. Five were reported as between students, two between school staff, two as staff-to-student, and no instances of student-to-staff transmission were reported. Of the nine suspected in-school transmissions, one was among grade TK-2 students, three were among grade 3-5 students, one was among grade 6-8 students, two were among teachers/school staff, and two were among special education students.

## Discussion

Findings from this descriptive analysis suggest that with proper site-specific safety plans, the 77 reopened Marin County schools were capable of safely conducting in-person learning with minimal in-school SARS-CoV-2 transmission during the 21-week observational period. While these are not causal associations, we observed that as the percentage of students returning to in-person learning increased, there was a concurrent decrease in countywide COVID-19 cases in Marin County. The increasing percentage of students returning to in-person learning appeared to serve as a protective factor against an increase in community-based COVID-19 cases, effectively mitigating an increase in countywide COVID-19 cases, as illustrated in Figures 1, 2.

For example, over the Thanksgiving holiday school break, spanning the period from November 23, 2020, to November 29, 2020, and the two-week winter holiday school break, from December 21, 2020, to January 3, 2021, countywide COVID-19 cases increased in the absence of in-person learning participants at the school sites. This observed trend could have been confounded by the increased travels, social gatherings, and mixing of different households. Also, it is important to note that these observational findings were noted when Marin County’s population immunization rate was at less than 1% [20] and COVID-19 vaccination eligibility did not yet cover educators and school staff, giving credence to the efficacy of cohesive implementation of mitigation strategies against SARS-CoV-2 spread within the school communities reopened for in-person learning, especially of strict adherence to universal face covering while on school grounds.

Additional noteworthy findings include the dramatic decline of percentage positivity and crude case rates in Marin County. Percentage positivity reached a peak of 11.1% [21] two months prior to the reopening of schools; it decreased to as low as 0.9% [21] during the surveillance period. The calculated crude case rate 61 days prior to school reopening was 89.9 (per 10,000) versus 35.89 (per 10,000) just 61 days into school reopening. These findings suggest that as more students returned to school for in-person instruction, community cases decreased. Of the 189 school-related cases (0.84%) among school staff and students, only nine were suspected to be in-school transmissions, with no instances of student-to-staff transmission. These results add to the evidence presented by the previously cited studies from schools in Utah and Wisconsin, concluding that children are more likely to contract COVID-19 in settings without face masking than in settings with adherence to it.

The more aggressive SARS-CoV-2 Delta variant, believed to be more contagious and rapidly transmissible, can also be contained by the mitigation strategies that are already known to be effective at minimizing the spread of COVID-19. The mental health status of educators [22] has been affected by concerns of safety with school operations and plays a fundamental element in the success of continued in-person instruction at school. A recently published case study [23] described an outbreak that occurred in late May-early June in a classroom due to inconsistent adherence to face mask use, with 12 out of 24 students contracting the SARS-CoV-2 Delta variant from their infected, asymptomatic, and unvaccinated teacher. The reported attack rate in that classroom was 50% with a risk correlation based on seating proximity to the unvaccinated, unmasked teacher. This reinforces the fact that strict adherence to mitigation strategies, specifically the use of face masks, is crucial to containing the viral spread. This becomes even more crucial when unvaccinated persons are involved. The preponderance of the evidence of the high transmissibility of the SARS-CoV-2 Delta variant highlights the need to support universal masking in the school settings, especially where a certain student population, still ineligible for the COVID-19 vaccine [24], remain vulnerable to infection. Consistent use of face masks when indoors is even more important, now that physical distancing is not as feasible to implement with a full return of students to the classrooms for in-person instruction. In addition to universal masking, there is increasing evidence that maximizing indoor ventilation and the use of HEPA air purifiers [25] add to the layer of protection against the spread of respiratory viruses, including SARS-CoV-2. The advent of pediatric vaccines adds another layer of arsenal in keeping COVID-19 minimal in the school settings as well as in the community.

Two important characteristics about Marin County include its demographics and the diversity among the County’s school districts. Non-Hispanic white and Latinx communities are the predominant ethnic groups residing in Marin County, at 71.1% [21] and 16.3% [20] of the total county population, respectively. The Latinx population accounts for 54.8% [21] of total COVID-19 cases and 32.8% [20] is accounted for by the White non-Hispanic population. School districts range from high-income to low-income. It is in impoverished communities that disparities and inequities are exacerbated. However, efforts implemented in the lower-income school districts, with over 70% [22] socioeconomically disadvantaged students, have reportedly been effective at mitigating the viral spread in the setting of in-person learning, as they implemented the 30-point safe school reopening plan [17] recommended by public health officials. Their success over the 21-week study period appears to have assisted in the reduction of COVID-19 cases in the harder-afflicted Latinx communities. A follow-up study on high schools will be an opportunity to conduct an important trends analysis in the near future as more high schools consistently reopen for in-person instruction. An analysis comparing COVID-19 trends in fall 2021 and the winter holidays will provide deep insights into the topic.

The strengths of this study include the collaboration of a credible variety of case investigators, contact tracers, public health officials, and epidemiologists from Marin County’s Public Health Division and administrators from the Office of Education. This 21-week long retrospective review of countywide COVID-19 cases and school COVID-19 data entails a longer study period than previously published studies of its kind. The study captured the trends of COVID-19 school data, including during two major fall/winter holidays. The sample size of the student population that returned to in-person school, and conducting the study at the county level, with collaboration between county epidemiologists, public health officials, and school administrators, represent the major strengths of the study. Similar studies from other counties in the nation have arrived at conclusions that mirror this study's results, in that the return of students to in-person school with adherence to proper mitigation strategies did not lead to an increase in COVID-19 cases in the community. In addition, similar international studies have reached the same conclusion that schools can safely operate during the pandemic, provided that safety protocols as advised by their public health officials are implemented. 

A limitation to this study is that no causal correlation was determined. The results of this study entail observational trends and descriptive analysis. Several confounding factors add limitations to the study, and they include social behaviors when public health orders were relaxed and tightened, and these may have contributed to the results. The socioeconomic contrast between the rich and poor areas of Marin County may also have contributed to the direction of the case rate. The disparity caused by this social determinant of health leads to overcrowding and multi-family dwelling scenarios, which then becomes a petri dish conducive for the rapid spread of respiratory viruses, including SARS-CoV-2. 

## Conclusions

Acquisition and transmission of COVID-19 among participants of in-person learning appears to be minimal among the 77 Marin County schools included in our 21-week surveillance period. There was a two-fold decrease in countywide COVID-19 cases as the percentage of students returning to in-person learning increased. The increases in COVID-19 community cases do not appear to be correlated with the increase in the percentage of in-person learning students. Suspected in-school SARS-CoV-2 transmission was minimal among these school settings. This surveillance study demonstrated that it is possible to mitigate COVID-19 outbreaks in in-person learning settings within school communities, besides helping to reduce the COVID-19 incidence rate in the community, even before COVID-19 vaccines were made accessible to educators and the pediatric population in the age bracket of 12 years and above. These findings can be applicable to the SARS-CoV-2 Delta variant and the winter season ahead.

It is incumbent on public health officials and school administrators to collaboratively develop an effective and consistent policy to stabilize school reopening prior to achieving fully-vaccinated status for the pediatric population. Public health prioritization of safe school reopening will improve the loss of education equity nationwide and worldwide for all students, and alleviate anxiety among stakeholders involved in the school reopening during a still rapidly-evolving pandemic. The scenario where pediatric COVID-19 vaccines will be accessible to the population in the age bracket of 5-11 years brings added hope in terms of the reopening of in-person instruction at schools globally.

## References

[1] Hoffman JA, Miller EA (2020). Addressing the consequences of school closure due to COVID-19 on children's physical and mental well-being (Epub ahead of print). World Med Health Policy.

[2] Cherubini V, Gohil A, Addala A, Zanfardino A, Iafusco D, Hannon T, Maahs DM (2020). Unintended consequences of coronavirus disease-2019: remember general pediatrics. J Pediatr.

[3] Li H, Yu G, Duan H, Fu J, Shu Q (2020). Changes in children's healthcare visits during coronavirus disease-2019 pandemic in Hangzhou, China. J Pediatr.

[4] Nicola M, Alsafi Z, Sohrabi C (2020). The socio-economic implications of the coronavirus pandemic (COVID-19): a review. Int J Surg.

[5] Asbury K, Fox L, Deniz E, Code A, Toseeb U (2021). How is COVID-19 affecting the mental health of children with special educational needs and disabilities and their families?. J Autism Dev Disord.

[6] Lambert JA, Trott K, Baugh RF (2020). An analysis of K-12 school reopening and its impact on teachers. J Prim Care Community Health.

[7] Falk A, Benda A, Falk P, Steffen S, Wallace Z, Høeg TB (2021). COVID-19 cases and transmission in 17 K-12 schools - Wood County, Wisconsin, August 31-November 29, 2020. MMWR Morb Mortal Wkly Rep.

[8] Laws RL, Chancey RJ, Rabold EM (2021). Symptoms and transmission of SARS-CoV-2 among children - Utah and Wisconsin, March-May 2020. Pediatrics.

[9] Zimmerman KO, Akinboyo IC, Brookhart MA (2021). Incidence and secondary transmission of SARS-CoV-2 infections in schools. Pediatrics.

[10] Macartney K, Quinn HE, Pillsbury AJ (2020). Transmission of SARS-CoV-2 in Australian educational settings: a prospective cohort study. Lancet Child Adolesc Health.

[11] Buonsenso D, De Rose C, Moroni R, Valentini P (2020). SARS-CoV-2 infections in Italian schools: preliminary findings after 1 month of school opening during the second wave of the pandemic. Front Pediatr.

[12] Lee EJ, Kim DH, Chang SH, Suh SB, Lee J, Lee H, Han MS (2021). Absence of SARS-CoV-2 transmission from children in isolation to guardians, South Korea. Emerg Infect Dis.

[13] Lee B, Raszka WV Jr (2021). COVID-19 in children: looking forward, not back. Pediatrics.

[14] Yung CF, Kam KQ, Chong CY (2020). Household transmission of severe acute respiratory syndrome coronavirus 2 from adults to children. J Pediatr.

[15] [Dataset]. MCOE, Marin County Office of Education (2021). MCOE, Marin County Office of Education. https://www.google.com/url?q=https://marinschools-my.sharepoint.com/:x:/g/personal/mgrant_marinschools_org/EaGaZHx7lFNCpF7D3eQGwYcB0UkVZjBUbm8cQzid88fwPQ&sa=D&source=editors&ust=1612820235627000&usg=AOvVaw21ZF1eSbkbMgqIjbADAf7u.

[16] [Dataset]. Marin HHS (2021). Marin HHS School Dashboard. https://coronavirus.marinhhs.org/schools.

[17] (2021). Marin Schools. 30-Point Plan for Safe School Reopening. https://www.marinschools.org/cms/lib/CA01001323/Centricity/Domain/154/FINAL%2012.8.20%20Marin%20County%20Schools%20Guidelines.pdf.

[18] (2020). Blueprint for a safer economy: education. https://www.cdph.ca.gov/Programs/CID/DCDC/Pages/COVID-19/Education.aspx.

[19] [Dataset]. Marin HHS (2021). Marin HHS School Tally Data Sheet. https://marincounty-my.sharepoint.com/:x:/r/personal/amcgrath_marincounty_org/_layouts/15/Doc.aspx?action=edit&sourcedoc=%7B512a0e3f-bdf7-40fe-9cc1-96cb230adf76%7D.

[20] Marin HHS (2021). Marin HHS COVID-19 vaccine data. https://coronavirus.marinhhs.org/vaccine/data.

[21] Marin HHS (2021). Marin HHS COVID-19 surveillance. https://coronavirus.marinhhs.org/surveillance.

[22] Shah K, Mann S, Singh R, Bangar R, Kulkarni R (2020). Impact of COVID-19 on the mental health of children and adolescents. Cureus.

[23] Lam-Hine T, McCurdy SA, Santora L, Duncan L, Corbett-Detig R, Kapusinszky B, Willis M (2021). Outbreak associated with SARS-CoV-2 B.1.617.2 (Delta) variant in an elementary school - Marin County, California, May-June 2021. MMWR Morb Mortal Wkly Rep.

[24] (2021). FDA. U.S. Food and Drug Administration. FDA approves first COVID-19 vaccine: approval signifies key achievement for public health. https://www.fda.gov/news-events/press-announcements/fda-approves-first-covid-19-vaccine.

[25] Hammond A, Khalid T, Thornton HV, Woodall CA, Hay AD (2021). Should homes and workplaces purchase portable air filters to reduce the transmission of SARS-CoV-2 and other respiratory infections? A systematic review. PLoS One.

